# Clinical experience of consolidative radiotherapy for localized metastatic non-small cell lung cancer who showed favorable tumor response after systemic treatment

**DOI:** 10.1007/s12672-024-00896-3

**Published:** 2024-03-06

**Authors:** Hakyoung Kim, Sun Myung Kim, Jeongeun Hwang, Dae Sik Yang

**Affiliations:** 1 Departments of Radiation Oncology, Korea University Guro Hospital, Korea University College of Medicine, 148 Gurodong-Ro, Guro-Gu, Seoul, 08308 Republic of Korea; 2 Department of Biomedical Research Center, Korea University Guro Hospital, Seoul, Republic of Korea; 3 Department of Medical IT Engineering, Soonchunhyang University, Asan, Chungcheongnam-do Republic of Korea

**Keywords:** Lung cancer, Metastasis, Radiotherapy, Local control, Survival

## Abstract

**Background:**

Our study has aimed to assess the effects of consolidative high-dose radiotherapy on clinical outcomes in patients with localized metastatic non-small cell lung cancer (NSCLC) who showed favorable tumor response after systemic treatment.

**Methods:**

We retrospectively reviewed the medical records of 83 patients with localized metastatic NSCLC, who received systemic therapy followed by consolidative local radiotherapy at the Korea University Guro Hospital between March 2017 and June 2022. In the current study, we defined localized metastatic disease as the presence of one to three metastatic sites at the time of diagnosis. And patients who showed favorable tumor response after systemic treatment, including oligo-progressive disease at the thoracic site which was amenable to curative high-dose local radiotherapy, were included. The planned total dose and fraction size mainly depended on the location of lesions.

**Results:**

The median follow-up time after consolidative radiotherapy was 16 months (range: 5–52 months). The overall 2-year progression-free survival rates were 81.4%. Of 83 patients, only four (4.3%), treated with intensity-modulated radiation therapy, showed an in-field local recurrence. Interestingly, only one patient experienced a local failure among the 20 patients who showed an oligo-progressive disease at the thoracic site on the tumor response evaluation after systemic treatment. Regarding treatment-related pulmonary toxicity, three patients with grade-3 and one patient with grade-4 radiation pneumonitis were presented.

**Conclusions:**

If the disease is sufficiently controlled and localized by systemic therapy, local consolidative radiotherapy is thought to improves local control rates with acceptable treatment-related toxicities in patients with localized metastatic NSCLC, especially those with oligo-progressive disease.

## Introduction

Remarkable advances in systemic agents have aroused more aggressive local therapy for various types of cancers [1–7]. Previous phase-II randomized clinical trials have shown that aggressive local consolidative high-dose radiotherapy to all lesions improves local control rates with acceptable treatment-related complications in patients with localized metastatic non-small cell lung cancer (NSCLC) [8–14].

While previous prospective studies included patients who showed controlled primary malignancies and no disease progression, most recently, the final analysis of the first randomized Consolidative Use of Radiotherapy to Block (CURB) Oligo-progression trial showed the beneficial effects of administering high-dose radiotherapy to sites of oligo-progression on prolonged progression-free survival (PFS) [15].

The current National Comprehensive Cancer Network (NCCN) guidelines recommend consolidative radiotherapy for selected patients with localized metastatic NSCLC, who have responded to prior chemotherapy and have a limited extrathoracic tumor burden. As a result of survival improvement due to the introduction of targeted and immuno-oncological agents, the importance of local treatment for residual lesions is gradually being emphasized. Despite these promising results, consolidative radiotherapy is not routinely performed in clinical practice. Against this background, our institution has been actively conducting consolidative radiotherapy for the above patients. Therefore, this study has aimed to assess the effects of consolidative high-dose radiotherapy on clinical outcomes in patients with localized metastatic NSCLC who showed favorable tumor response after systemic treatment.

## Methods

### Patients

After the approval of the institutional review board (no. 2022GR0196), we retrospectively reviewed the medical records of 83 patients, who received systemic therapy followed by consolidative local radiotherapy at Korea University Guro Hospital between March 2017 and June 2022. The inclusion criteria were as follows: (1) having histologically confirmed NSCLC; (2) having stage IV disease with one to three metastatic sites; (3) showing partial response or stable disease or oligo-progressive disease on tumor response evaluation after systemic treatment; (4) having an Eastern Cooperative Oncology Group performance status of 0–2; and (5) having physiologic suitability to undergo radiotherapy. Therapeutic decisions were made on an individual patient basis at a multidisciplinary lung cancer conference. In the current study, we defined localized metastatic disease as the presence of one to three metastatic sites at the time of diagnosis [10–13]. And patients who showed favorable tumor response after systemic treatment, including oligo-progressive disease at the thoracic site which was amenable to curative high-dose local radiotherapy, were included.

### Treatment scheme

Consolidative radiotherapy was administered to up to three sites that remained active on the positron emission tomography-computed tomography (PET-CT) data. The planned total dose and fraction size depended on the location of lesions. With respect to the target delineation of lung lesion, the gross tumor volume (GTV) was delineated under the lung window setting. The internal target volume (ITV) was delineated following four-dimensional CT with special regard to the patient’s respiratory motion. The clinical target volume (CTV) was generated with a 5-mm expansion of the GTV-ITV in all directions and then modified according to the adjacent normal anatomic structures. The planning target volume (PTV) was generated with a 5-mm expansion of the CTV. Regarding treatment planning, a hypofractionated regimen was administered, whenever possible, to patients with NSCLC. Stereotactic ablative radiation therapy (SABR) with a total dose of 60 Gy in four fractions was administered to patients with NSCLC of small-sized (≤ 4 cm) and peripherally located tumors. For patients who received intensity-modulated radiation therapy (IMRT), different dose-fractionation schedules were planned to deliver 60 Gy in 20 fractions over 4 weeks or 50–70 Gy in 5–10 fractions over 1–2 weeks, respectively. When the shortest distance between the CTV margin and the esophagus was ≥ 1.5 cm, 5–10 fractions were preferred to 20 fractions.

With respect to the target delineation of bone lesion, the GTV were delineated under the bone window setting. The CTV were generated with 5 mm expansion of the GTV in all directions, which were then modified according to the adjacent anatomic structures. The simultaneous integrated boost (SIB) technique was applied to deliver different dose levels to GTV and CTV within a single treatment fraction. SABR was conducted for spine lesions with total dose of 32/24 Gy in four fractions. In cases of IMRT, two different dose-fractionation schedules were used for the delivery of 35/30 Gy in 5 fractions or 30/25 Gy in 5 fractions, respectively, depending on whether the lesion contains joint areas. In patients with other lesions, IMRT was administered with a total dose of 35–45 Gy in 5–15 fractions, depending on the distance between the CTV margin and normal organ, with reference to the PET-CT data.

The prescription guideline was to deliver at least 97% of the prescribed dose to 95% of the PTV. The minimum and maximum doses to 1 cc of PTV were 95% and 107%, respectively. The percentage lung volume that received ≥ 20 Gy was to be kept ≤ 35%, and the mean lung dose was ≤ 20 Gy. The maximum doses to the spinal cord and esophagus were not to exceed 45 Gy and 60 Gy, respectively, satisfying the dose-volume constraints of normal organ.

### Surveillance

We assessed tumor responses using contrast-enhanced chest/abdomen/pelvis computed tomography (CT) scans for every two cycles of systemic therapy and at completion of therapy to assess disease progression during follow-up. The revised Response Evaluation Criteria in Solid Tumors guidelines (version 1.1) was used to evaluate tumor responses. Treatment-related complications were evaluated using the Common Terminology Criteria for Adverse Events (version 4.03).

### Statistical analysis

PFS was defined as the time from the initiation of systemic therapy to the date of the first documentation of disease progression or the latest documented follow-up visit after receiving consolidative radiotherapy. Overall survival (OS) was defined as the time from the initiation of systemic therapy to the date of death from any cause or the latest documented follow-up visit. The 2-year PFS and OS rates were calculated using the Kaplan–Meier method and compared using the log-rank test. Statistical significance was set at p < 0.05. Statistical analyses were performed using IBM SPSS Statistics for Windows (version 24.0; IBM Corporation, Armonk, NY, USA).

## Results

### Baseline characteristics

The clinical characteristics of the patients are summarized in Table 1. the median age of the study population was 70 years (range: 36–91 years). Most patients were males (67.5%) and had the adenocarcinoma histological type (73.5%). Most patients had one metastatic site (61.4%) at the time of diagnosis and pleura was the most common site of distant metastasis. Among patients with stage-IV disease, 50 (60.2%) were treated with platinum-doublet chemotherapy and 33 (39.8%) were treated with targeted agents; tyrosine kinase inhibitor of the epidermal growth factor receptor (EGFR-TKI). Among 83 patients, 38 patients (45.8%) were treated with immuno-oncologic agents targeting programmed death-1 (PD-1) and programmed death-ligand 1 (PD-L1). Among those, 15 patients (15/38, 39.5%) were treated with combined immune-chemotherapy as an initial treatment. Of these patients, 23 (27.7%) showed partial response, 40 (48.2%) showed stable disease, and 20 (24.1%) showed an oligo-progressive disease on the tumor response evaluation carried after administering systemic therapy. The median time from the start of systemic treatment to consolidative radiotherapy was 16 months (range: 3–39 months).

**Table 1:** Clinical characteristics (*N* = 83)

Characteristics	NSCLC (*N* = 83)	
	Number	%
Age [years; median (range)]	70 (36–91)	
Sex		
Female	27	32.5
Male	56	67.5
Smoking Status		
Never smoker	44	53.0
Current or Ex-smoker	39	47.0
ECOG performance status		
0–1	81	97.6
2	2	2.4
Histology		
Adenocarcinoma	61	73.5
Squamous cell carcinoma	20	24.1
Others	2	2.4
Prior treatment		
Platinum-doublet chemotherapy	50	60.2
Target agents	33	39.8
Number of metastatic sites		
1	51	61.4
2–3	32	38.6
Immuno-oncologic agent treatment		
No	45	54.2
Yes	38	45.8
Response to systemic treatment		
Partial response	23	27.7
Stable disease	40	48.2
Oligo-progressive disease	20	24.1
Radiotherapy technique		
IMRT	46	55.4
SBRT	37	44.6

*NSCLC* Non-Small Cell Lung Cancer, *SCLC* Small Cell Lung Cancer, *ECOG* Eastern Cooperative Oncology Group, *IMRT* Intensity Modulated Radiation Therapy, *SBRT* Stereotactic Body Radiation Therapy

### Patterns of failure

**Fig. 1:**
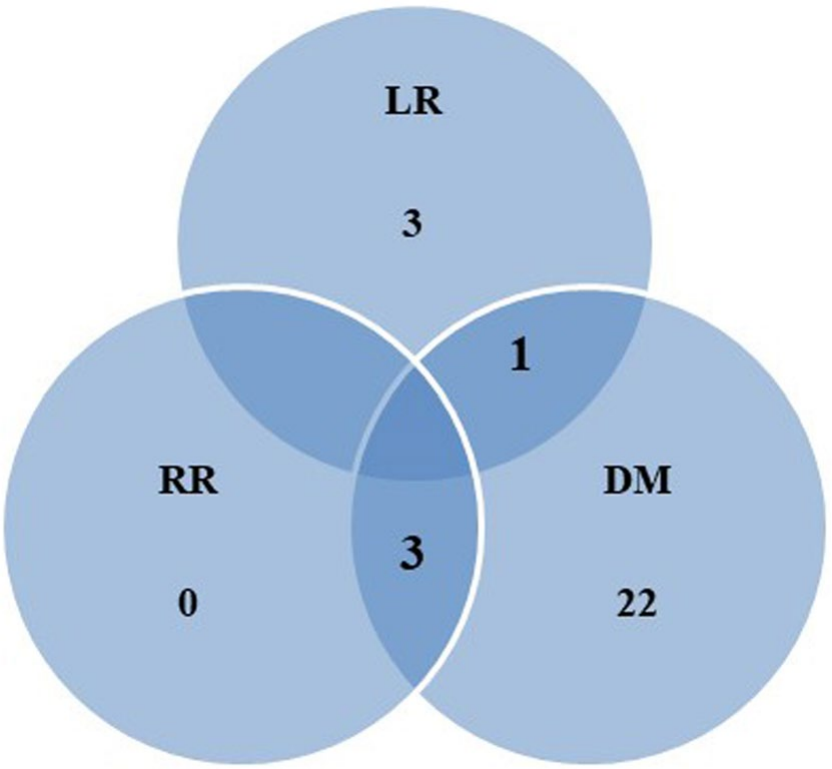
Patterns of failure in localized metastatic NSCLC groups. *NSCLC* non-small cell lung cancer, *LR* local recurrence, *RR* regional recurrence, *DM* distant metastasis

The median follow-up time after consolidative radiotherapy was 16 months (range: 5–52 months). The failure patterns are shown in Fig. 1. Distant metastasis was the most common type of recurrence (26/83 [31.3%]). Distant metastasis solely, locoregional recurrence solely, and both occurred in 22, 3, and 4 patients, respectively. The most common site of distant metastasis was the lung (10 patients), followed by the brain (8 patients), and bone (7 patients). Regarding radiotherapy, in-field local recurrences occurred in four patients (4/83 [4.8%]). Only one patient experienced a local failure among the 20 patients who showed an oligo-progressive disease at the thoracic site on the tumor response evaluation after systemic treatment.

### Survival outcomes and treatment-related complications

**Fig. 2:**
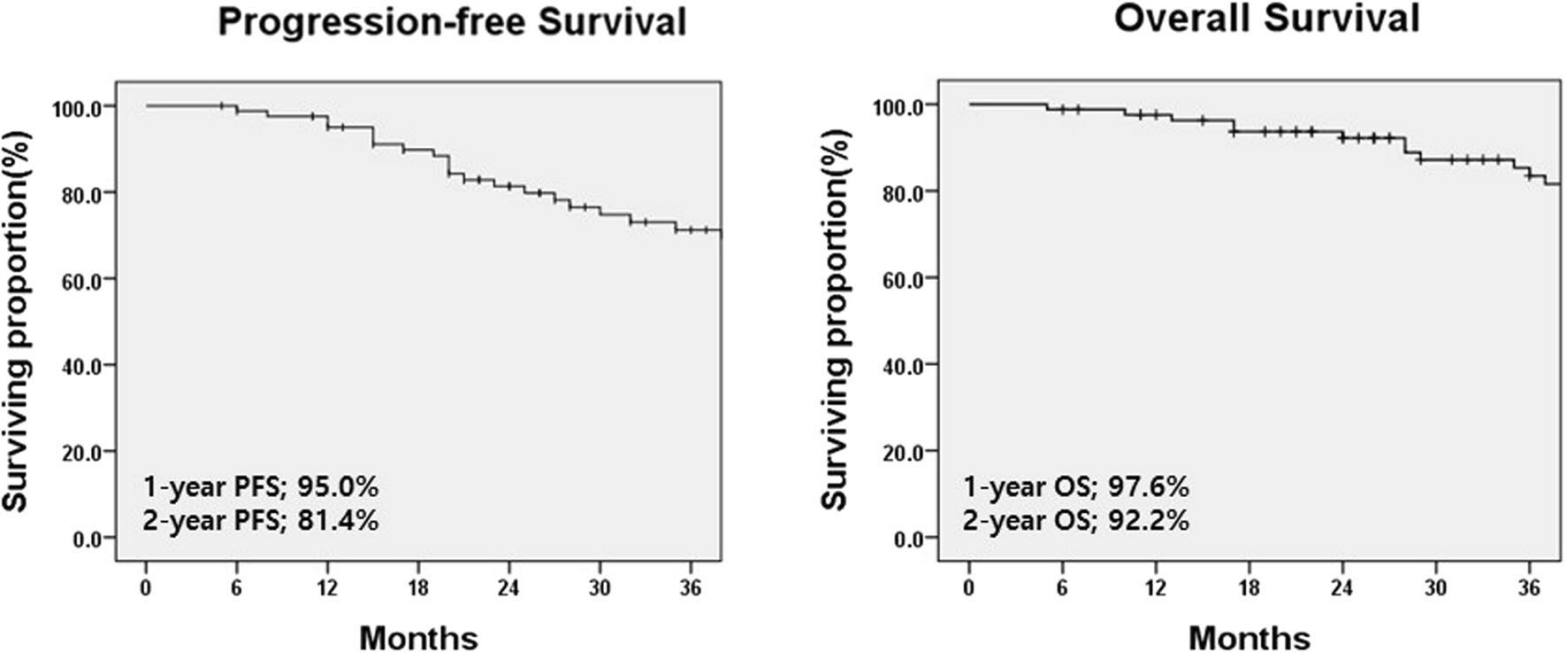
Survival curves in patients with localized metastatic non-small cell lung cancer treated with consolidative aim. *PFS* Progression-free survival, *OS* Overall survival

The overall 2-year PFS and OS rates were 81.4% and 92.2%, respectively (Fig. 2). In univariate analysis (Table 2), patients with adenocarcinoma histologic type showed a tendency for better PFS compared to those with non-adenocarcinoma histologic type (*p* = 0.064). Similarly, patients treated with prior targeted agents showed a tendency for better PFS than those treated with prior platinum-doublet chemotherapy (p = 0.091).

**Table 2:** Univariate analysis for factors associated with survival outcomes (*N* = 83)

Variable	2-year PFS rate (%)	p-value	2-year OS rate (%)	p-value
Gender		0.068		0.032
Female	95.8		100	
Male	74.1		88.4	
Smoking Status		0.616		0.126
Never smoker	82.9		100	
Current or Ex-smoker	79.3		82.9	
Histology		0.064		0.022
Adenocarcinoma	85.6		96.3	
Non-adenocarcinoma	68.1		80.0	
Number of metastatic sites		0.184		0.676
1	85.1		93.8	
2–3	75.2		89.7	
Prior treatment		0.091		0.018
Platinum-doublet chemotherapy	76.7		86.7	
Target agents	87.6		100	
Immuno-oncologic agent		0.702		0.526
No	80.6		93.2	
Yes	82.0		90.9	
Response to systemic treatment		0.956		0.446
Partial response/ Stable disease	83.6		91.5	
Oligo-progressive disease	74.7		94.4	

*PFS* Progression-free survival, *OS* Overall survival

Female gender (p = 0.032), adenocarcinoma histologic type (p = 0.022), and prior treatment with targeted agents (p = 0.018) were significantly associated with OS on univariate analysis. Interestingly, the response to initial systemic treatment was not significantly associated with either PFS or OS (p = 0.956 and p = 0.446, respectively).

Regarding treatment-related pulmonary toxicity, the incidence of grade-2 radiation pneumonitis, requiring oral steroid medication, was 24.1% (20/83). Three patients with grade-3 and one patient with grade-4 radiation pneumonitis were presented. The patient who developed grade-4 complications was a male with a history of smoking. In addition, he had an underlying lung disease (specifically, idiopathic pulmonary fibrosis) and showed impaired pulmonary function (the pre-IMRT diffusing capacity of the lung for carbon monoxide was 55%). Severe radiation pneumonitis occurred 2 months after the completion of IMRT.

## Discussion

Regarding oligo-progression, the NCCN guidelines state that consolidative local radiotherapy at oligo-progressive sites may extend the duration of the current systemic therapy benefits. Most recently, a final analysis of the first and largest randomized CURB trials [15] showed the impact of SABR in oligo-progressive metastatic sites on prolonged PFS (median PFS, 10 months in the SABR arm vs. 3.2 months in the standard-of-care arm, P = 0.002). Accordingly, this study included 20 patients who showed oligo-progressive disease at the thoracic site on the tumor response evaluation after systemic treatment, which was amenable to curative high-dose local radiotherapy according to our favorable clinical experience. Interestingly, only one patient experienced a local failure among the 20 patients who showed an oligo-progressive disease on the tumor response evaluation carried after administering systemic treatment. And other oligo-progressive sites were well maintained without local progression even without changing the current line of systemic treatment.

Consolidative radiotherapy has several roles in treating patients with localized metastatic NSCLC. The initial results of the SABR-COMET trial showed a remarkable improvement in PFS [1] and recent follow-up data [14] showed that the impact of SABR on PFS (5-year PFS, not reached in arm 1 and 17.3% in arm 2, P = 0.001) was larger than that in the initial results and was durable over time. Moreover, another randomized phase-II trial was conducted to asses local consolidative therapy (LCT) in treating stage-IV NSCLC, and recent follow-up data [9, 10] confirmed that LCT significantly improved PFS (median PFS: 4.4 months vs. 14.2 months). The current NCCN guidelines recommend consolidative radiotherapy in patients with low-bulk extrathoracic diseases, who have favorably responded to the initial systemic therapy. In the current chemo-immunotherapy use era, hypofractionated radiotherapy such as SABR, which can exert immune-modulating effects, is more actively recommended than IMRT. However, to date, no data have been reported on the optimal treatment regimen, order, or toxicity.

This study had some limitations. First, it had a retrospective nature; thus, a selection bias might have occurred. Second, the sample size was too small to demonstrate clinical significance. Nonetheless, we have aimed to evaluate the effect of consolidative high-dose radiotherapy on local control rates and survival outcomes in patients with localized metastatic NSCLC, especially focusing on oligo-progressive disease. This study showed promising results that are consistent with those of previous studies. The overall 2-year PFS rates were 81.4% in the localized metastatic NSCLC group. Among all patients, only four (4/83 [4.8%]), who were treated with IMRT, showed an in-field local recurrence. Especially, patients with adenocarcinoma histologic type and treated with prior targeted agents showed a tendency for better PFS and significantly improved OS. However, the response to initial systemic treatment was not significantly associated with either PFS or OS (p = 0.956 and p = 0.446, respectively).

## Conclusions

If the disease is sufficiently controlled and localized by systemic therapy, local consolidative radiotherapy is thought to improves local control rates with acceptable treatment-related toxicities in patients with localized metastatic NSCLC, especially those with oligo-progressive disease.

## Data Availability

The datasets used and/or analyzed in the current study can be obtained from the corresponding author upon reasonable request.

## Abbreviations

NSCLC: Non-small cell lung cancer
CURB: Consolidative use of radiotherapy to block
PFS: Progression-free survival
NCCN: National comprehensive cancer network
PET-CT: Positron emission tomography-computed tomography
GTV: Gross tumor volume
ITV: Internal target volume
CTV: Clinical target volume
PTV: Planning target volume
SABR: Stereotactic ablative radiation therapy
IMRT: Intensity-modulated radiation therapy
OS: Overall survival
LCT: Local consolidative therapy

## References

[1] Palma DA, Olson R, Harrow S, Gaede S, Louie AV, Haasbeek C (2019). Stereotactic ablative radiotherapy versus standard of care palliative treatment in patients with oligometastatic cancers (SABR-COMET): a randomised, phase 2, open-label trial. Lancet.

[2] Chawla S, Chen Y, Katz AW, Muhs AG, Philip A, Okunieff P (2009). Stereotactic body radiotherapy for treatment of adrenal metastases. Int J Radiat Oncol Biol Phys.

[3] Gerszten PC, Mendel E, Yamada Y (2009). Radiotherapy and radiosurgery for metastatic spine disease: what are the options, indications, and outcomes?. Spine.

[4] Holy R, Piroth M, Pinkawa M, Eble MJ (2011). Stereotactic body radiation therapy (SBRT) for treatment of adrenal gland metastases from non-small cell lung cancer. Strahlenther Onkol.

[5] Milano MT, Katz AW, Muhs AG, Philip A, Buchholz DJ, Schell MC (2008). A prospective pilot study of curative-intent stereotactic body radiation therapy in patients with 5 or fewer oligometastatic lesions. Cancer.

[6] Méndez Romero A, Wunderink W, Hussain SM, De Pooter JA, Heijmen BJ, Nowak PC (2006). Stereotactic body radiation therapy for primary and metastatic liver tumors: a single institution phase i–ii study. Acta Oncol.

[7] Méndez Romero A, de Man RA (2016). Stereotactic body radiation therapy for primary and metastatic liver tumors: from technological evolution to improved patient care. Best Pract Res Clin Gastroenterol.

[8] Schanne DH, Heitmann J, Guckenberger M, Andratschke NHJ (2019). Evolution of treatment strategies for oligometastatic NSCLC patients—a systematic review of the literature. Cancer Treat Rev.

[9] Gomez DR, Blumenschein GR, Lee JJ, Hernandez M, Ye R, Camidge DR (2016). Local consolidative therapy versus maintenance therapy or observation for patients with oligometastatic non-small-cell lung cancer without progression after first-line systemic therapy: a multicentre, randomised, controlled, phase 2 study. Lancet Oncol.

[10] Gomez DR, Tang C, Zhang J, Blumenschein GR, Hernandez M, Lee JJ (2019). Local consolidative therapy vs maintenance therapy or observation for patients with oligometastatic non-small-cell lung cancer: long-term results of a multi-institutional, phase II, randomized study. J Clin Oncol.

[11] Iyengar P, Wardak Z, Gerber DE, Tumati V, Ahn C, Hughes RS (2018). Consolidative radiotherapy for limited metastatic non-small-cell lung cancer: a phase 2 randomized clinical trial. JAMA Oncol.

[12] Conibear J, Chia B, Ngai Y, Bates AT, Counsell N, Patel R (2018). Study protocol for the SARON trial: a multicentre, randomised controlled phase III trial comparing the addition of stereotactic ablative radiotherapy and radical radiotherapy with standard chemotherapy alone for oligometastatic non-small cell lung cancer. BMJ Open.

[13] Palma DA, Salama JK, Lo SS, Senan S, Treasure T, Govindan R (2014). The oligometastatic state—separating truth from wishful thinking. Nat Rev Clin Oncol.

[14] Palma DA, Olson R, Harrow S, Gaede S, Louie AV, Haasbeek C (2020). Stereotactic ablative radiotherapy for the comprehensive treatment of oligometastatic cancers: long-term results of the SABR-COMET phase II randomized trial. J Clin Oncol.

[15] Tsai CJ, Yang JT, Guttmann DM, Shaverdian N, Eng J, Yeh R (2022). Final analysis of consolidative use of radiotherapy to block (CURB) oligoprogression trial—a randomized study of stereotactic body radiotherapy for oligoprogressive metastatic lung and breast cancers. Int J Radiat Oncol Biol Phys.

